# A Comparison of Maternal Health Status and Weight-Related Cognitions, Behaviors, and Home Environments by Race/Ethnicity

**DOI:** 10.3390/nu12113592

**Published:** 2020-11-23

**Authors:** Colleen L. Delaney, Kim Spaccarotella, Carol Byrd-Bredbenner

**Affiliations:** 1 Department of Nutritional Science, Rutgers University, New Brunswick, NJ 08854, USA; bredbenner@sebs.rutgers.edu; 2 Department of Biological Sciences, Kean University, Union, NJ 07083-7131, USA; kspaccar@kean.edu

**Keywords:** race/ethnicity, mothers, health, nutrition, home environment, behavior

## Abstract

This cross-sectional study compared weight-related cognitions, behaviors, and home environments of 568 mothers of young children (ages 2 to <9 years) by racial/ethnic group. Maternal health status was good and did not differ by race/ethnicity. Mothers were somewhat confident in their ability to promote healthy physical activity and eating behaviors in their children, with White and Asian mothers having greater confidence than Hispanic mothers. Mothers had low physical activity, with Hispanic mothers getting more sedentary screentime than White and Asian mothers. Mothers’ dietary intake did not differ. Modeling of healthful behaviors was more frequent in White than Hispanic mothers. Asian mothers tended to use non-recommended feeding patterns more than White, Hispanic, and Black mothers. Children’s physical activity and screentime did not differ by race/ethnicity. Asian children tended to drink less sugar-sweetened beverages and more milk than counterparts. All reported frequent family meals, with Hispanic mothers reporting more family meals eaten in less healthful locations. Household food environments did not differ. However, White mothers reported greater access to physical activity space and supports than Hispanic mothers. Race/ethnicity may link with maternal weight-related cognitions, behaviors, and home environments and thus can help inform the development of interventions tailored by race/ethnicity.

## 1. Introduction

Obesity rates in the United States have more than doubled since 1970 [1,2,3]. This increase is even more drastic for minorities: obesity and overweight prevalence rose 120% for Blacks and Hispanics over the past three decades [4]. According to the 2011–2012 National Health and Nutrition Examination Survey (NHANES) data, 69% of adults are obese or overweight, and this percent rises to 78% for Hispanic adults and 76% for Black adults [5,6]. Among children living in the U.S., Hispanic children have the highest rates of overweight among all racial and ethnic groups, with 39% being overweight or obese versus 29%, 35%, and 20% of non-Hispanic White, Black, and Asian children [5]. This disproportion is even more profound among preschool-aged Hispanic children in the U.S., 22% of whom are obese compared to 8% of all other U.S. preschool-aged children [7].

The high and rising prevalence of obesity is alarming, especially considering the lifelong impact that excess body fat has on physical and mental health [6,8]. Some of the emotional and mental health outcomes associated with overweight and obesity include anxiety disorders, depression, low self-esteem, and increased stress, perhaps caused by the discrimination, bullying, and teasing experienced by many who are overweight [3,4,9,10,11]. Obesity also affects physical health—it can affect almost all of the organ systems and is associated with hypertension, chronic inflammation, and cardiovascular, pulmonary, gastrointestinal, renal, musculoskeletal, and endocrine complications, in addition to non-alcoholic fatty liver disease, sleep apnea, asthma, early menarche, malnutrition and nutritional deficiencies, and premature mortality [3,4,9,11,12,13,14,15,16,17].

The health complications of overweight and obesity are costly to society, with lifetime direct medical costs estimated at $19,000 per person [18]. National health care expenditures related to obesity and overweight in adults range from $98 to $129 billion dollars per year [19]. Indirect costs are even higher; these are related to lost productivity caused by morbidity, disability, or mortality due to comorbidities, such as type 2 diabetes mellitus, coronary heart disease, hypertension, certain cancers, and musculoskeletal diseases [20,21,22,23]. The costs of obesity and its associated comorbidities make a clear point for the need for enhanced public health efforts.

In recent years, significant research has focused on identifying environmental and personal factors associated with increased obesity risk and developing interventions to ameliorate these factors [24]. Interventions tailored to the needs and interests of specific target audiences are associated with greater acceptance and application of intervention messages [25]. Few interventions have directly considered racial/ethnic differences [26,27] even though these groups represent a significant proportion of the U.S. population and are disproportionately affected by obesity and overweight [5,27,28,29].

The limited research examining differences among racial/ethnic groups regarding weight-related practices makes it difficult to develop interventions targeted to the needs and interests of these groups. Available data do suggest that some weight-related practices, such as parental feeding practices and family mealtime practices, differ by race and ethnicity [30,31,32], and thereby indicate a more thorough examination of weight-related differences by race/ethnicity is warranted. This examination is particularly important considering that racial and ethnic minorities often face a disproportionate incidence of poor health and have the potential to benefit from health interventions targeted to their needs and sensibilities [33]. Thus, this cross-sectional study aimed to compare the health status and weight-related cognitions, behaviors, and home environments of mothers of young children (ages 2 to <9 years) by race/ethnicity. A second purpose was to use study findings to identify specific topics to highlight in nutrition and obesity prevention interventions targeted to mothers of young children.

## 2. Materials and Methods

This study was approved by the Institutional Review Board at the lead author’s university (approval #11-294Mc). The current study is a secondary analysis of the HomeStyles randomized controlled trial baseline “**H**ome **O**besogenicity **M**easure of **E**nvironment**S**” (HOMES) survey data. HomeStyles uses a social ecological framework to target improvements in maternal weight-related interpersonal cognitions and behaviors and characteristics of home environments to promote optimal child health, growth, and body weight [34]. HomeStyles participants completed the baseline HOMES survey prior to being randomized into experimental or control groups [34]. Results of the HomeStyles study have been reported elsewhere for a sub-set of the participants [24,35].

### 2.1. Sample

The study sample was recruited using materials in English and Spanish that invited parents to join a program to help them “build even happier, healthier, safer families”. Recruitment was conducted using in-person methods (e.g., recruitment at farmers markets, county fairs, community events), printed flyers posted at various locations (e.g., gyms, grocery stores, doctors’ offices), electronic announcements sent to community organizations serving families (e.g., religious groups, daycares/schools, after school care, summer camps, extracurricular programs, English as Second Language programs), workplaces, and by a research participant recruitment company. Eligible participants who gave informed consent and completed the baseline HOMES survey received a $15 stipend.

Of the 5494 individuals who responded to the recruitment notices, 5277 completed the study screener. Participants were removed from the sample if they did not complete the screener (*n* = 217), did not consent (*n* = 405), did not meet all inclusion criteria for the current study (i.e., aged 20–45 years of age, at least 1 child aged 2 to <9 years, primary household food gatekeeper [i.e., made all or most decisions related to family food choices], lived in the catchment area of NJ or AZ; *n* = 3343), did not complete the survey (*n* = 862), or provided implausible answers (e.g., gave same answer to all questions in a series; *n* = 34). Due to low response rates, fathers (*n* = 49) and participants reporting their race as mixed (*n* = 12), Alaskan Native or Pacific Islander (*n* = 2), and Native American or American Indian (*n* = 2) were excluded from the current study. The final analytic sample was 568 mothers.

### 2.2. Instrument

Development of the baseline HOMES survey is reported in detail elsewhere [24,36]. In brief, development began with a comprehensive review of the literature to determine cognitions, behaviors, and environmental factors associated with weight in children and parents and location of validated scales for assessing these factors [24,36]. When multiple measures were identified, experts in nutrition and survey methodology reviewed each measure to identify those that were most relevant, reliable, valid, easiest to administer and score, and had the lowest participant time burden [36]. For factors lacking a pre-existing scale or one that would fit the needs of this study, items were developed following Redding et al.’s recommendations for a sequential approach to measurement development [37]. These items, and those that were heavily modified from their original format, were reviewed by subject matter experts to ensure clarity and content validity [38,39]. In addition, items created de novo underwent cognitive testing with participants having similar characteristics to the study sample but who did not complete the baseline HOMES survey [40,41]. Subsequently, all measures were combined into a single online survey and pretested by 48 individuals eligible for, but not included in, the baseline HOMES survey. The pretest was conducted to ensure the functionality of the online survey, to determine completion time, and to ensure the protocols for scoring the scales were accurate. A field test with 550 individuals with the same characteristics as, but not participating in, the HomeStyles study was conducted to determine internal consistency, scale unidimensionality, and participant satisfaction. The panel of experts reviewed the final HOMES survey and outcomes to confirm its appropriateness to study purpose and audience [24,36,42].

In the baseline administration of the survey, which yielded the data reported here, mothers were asked to report demographic characteristics, food insecurity risk, health status, weight-related cognitions and behaviors, and child feeding practices. Mothers also reported the health status, physical activity, and eating behaviors for one of their children. If a mother had more than one child between the ages of 2 and <9 years, she was instructed to report on the child whose birthday was closest to noon on June 1 (a randomly selected date and time). Home environment assessments included family mealtime behaviors and home food and physical activity environment.

#### 2.2.1. Independent Variables

Mothers reported on these demographic characteristics: age, highest education level achieved, race/ethnicity, family affluence, number of parents in the household, and employment status (does not work, works part time, works full time). Race/ethnicity was indicated as White, Hispanic, Black, Asian, Native American or American Indian, and/or Alaskan Native or Pacific Islander. The Family Affluence Scale, considered a reliable indicator of family socioeconomic status, is a 10-point scale (0 to 9), with higher scores indicating greater affluence [43,44]. Hager’s 2-item scale was assessed food insecurity risk [45].

#### 2.2.2. Dependent Variables

The Centers for Disease Control and Prevention Health-Related Quality of Life Scale 5-point (poor to excellent) health rating item was used to measure both maternal and child health status [46,47]. Maternal cognitions (i.e., mothers’ confidence in the ability to promote children’s physical activity and healthy eating) were evaluating using 5-point Likert scales (strong disagree to strongly agree) developed and validated for the HOMES survey [24,36,42].

Mothers’ modeling behaviors for physical activity and healthy eating also were assessed using scales developed and validated for the HOMES survey [24,36,42]. The Maternal Modeling of Physical Activity scale assessed the days per week mothers engaged in co-play with their children. The Maternal Modeling of Healthy Eating scale assessed the importance mothers placed on this behavior using a 5-point Likert agreement scale (strongly disagree to strongly agree). The 3-item HOMES Physical Activity Questionnaire evaluated maternal and child physical activity level [24,42,48] by assessing frequency of engaging in walking, moderate, and vigorous activity. Minutes per day of screentime (use of television, movies, videos, computers) served as an indicator of mother and child sedentary activity. The Block Fruit/Vegetable/Fiber Screener, a food frequency scale, estimated mothers’ intake daily of fruits and vegetables and children’s daily intake of fruit/vegetable juice [49,50,51]. The HOMES Drinks Intake Screener estimated mother and child daily servings of sugar-sweetened beverages (i.e., soft, fruit, tea, coffee, and energy drinks) as well as children’s milk intake [24,52,53]. Mothers’ child feeding practices were evaluated using 3 Likert-type agreement scales that assessed use of food to reward children’s healthy eating, use of pressure to compel children to eat, and control of children’s food intake choices [42,54,55,56,57,58,59,60].

The home food environment characteristics evaluated were meals per week the family ate together and days each week family meals were eaten in circumstances associated with healthier meals (dining table) or less healthy meals (car, fast food restaurant, in front of the television) [61,62,63,64,65]. Household availability of fruits/vegetables and sugar-sweetened beverages was assessed using food frequency questionnaires that determine servings available daily per person in the household [24]. Scales from the Hop-Up questionnaire were used to assess the home physical environment [66,67]. This questionnaire assesses the space and supports for physical activity that the family has available inside the home, in the outdoor/yard area right outside the home, and in the neighborhood.

### 2.3. Data Analysis

Mothers were divided into four major race/ethnicity categories based on survey responses (White, Black, Hispanic, and Asian). Native American or American Indian, Alaskan Native or Pacific Islander, and mixed-race participants were excluded due to very low enrollment numbers. Descriptive statistics (e.g., means, standard deviations, confidence intervals) were calculated to describe the sample characteristics and performance on each measure. Analysis of variance (ANOVA) and Tukey post-hoc tests were conducted to determine differences among racial/ethnic categories. Due to the numerous comparisons planned, the Benjamini–Hochberg procedure was implemented at a 5% rate for two-tailed tests, yielding a probability level for the main effects (ANOVA) set at *p* ≤ 0.02 to reduce the risk of type I errors [68]. Post-hoc probability was set at *p* < 0.05. Partial eta-squared values were calculated to indicate effect size of significant ANOVA comparisons, with effect sizes of 0.01, 0.06, and 0.14 indicating small, medium, and large effects, respectively [69]. All analyses were computed using SPSS software version 27.0 (IBM Corporation, Chicago, IL, USA).

## 3. Results

Mothers were 32.73 ± 5.55SD years old and were predominately White (60%) and Hispanic (26%), with fewer mothers describing themselves as Black (8%) or Asian (6%). As shown in Table 1, compared to other racial/ethnic groups, Hispanic mothers were significantly younger and Asian mothers had a significantly higher education level. Most mothers had at least some college education (86%); few mothers reported having a high school diploma or less (14%). Family affluence was moderate, with White mother having a significantly higher affluence level than both Hispanic and Black mothers. Food insecurity risk was below the scale midpoint and did not differ across racial/ethnic groups. White and Asian mothers were more likely to be in dual parent households than other comparison groups. All groups were similar in their employment status, with the mean scores indicating most worked part time. Effect size was small for all demographic characteristics that differed significantly.

Maternal health status tended to be good to very good and did not differ by racial/ethnic group (Table 2). An examination of mothers’ cognitions revealed that all groups of mothers were somewhat confident in their ability to promote healthy physical activity and eating behaviors in their children. However, White and Asian mothers tended to have significantly more confidence in their ability to promote healthy child physical activity and eating behaviors than Hispanic mothers with small effect sizes.

All mothers had low physical activity scores, scoring between 12 and 16 on a scale ranging from 0 to 42. Time mothers spent in sedentary screentime activities ranged from approximately 4 to 7 h daily, with Hispanic mothers getting significantly more daily screentime than both White and Asian mothers. Mothers averaged 4 to 5 servings of fruits and vegetables daily and drank less than one serving of sugar-sweetened beverages. Maternal modeling of physical activity and healthy eating scores were moderate, with White mothers engaging in these modeling behaviors significantly more than Hispanic mothers. All significant differences in maternal behaviors had small effect sizes.

Mothers tended to be fairly neutral with regard to rewarding children with food, pressuring them to eat, and controlling their food intake amounts, with Asian mothers scoring higher on all of these child feeding measures. In fact, Asian mothers tended to score significantly higher on all child feeding practices than both White and Hispanic mothers. Hispanic and White mothers, on the other hand, scored significantly lower on controlling children’s food intake amounts than both Black and Asian mothers, with a medium effect size.

Mothers reported that their children’s health status was good to excellent, with White mothers reporting significantly higher child health than both Hispanic and Asian mothers (Table 3). Children had moderate levels of physical activity and got an average of approximately 5 h of screentime daily, with no differences across groups. Sugar-sweetened beverage intake was low. However, Asian children had significantly fewer servings daily than both Black and Hispanic children. Asian children had significantly more daily servings of milk than all other groups, with a small effect size. White children consumed significantly less 100% fruit juice than both Hispanic and Black children; effect size was small.

As shown in Table 4, all groups of mothers agreed that family meals were important and reported eating approximately two meals each day as a family. Locations where meals were eaten differed significantly, with Hispanic mothers reporting more family meals eaten in the car than all other groups. In addition, Hispanic moms reported more family meals eaten at fast food restaurants and while watching television, and fewer eaten at a dining table than White mothers. All family mealtime significant differences had small effect sizes.

Household food availability indicated each person in the household had access to 5 to 6 servings of fruits and vegetables daily and approximately one-quarter of a serving of sugar-sweetened beverages. Groups of mothers did not differ in terms of household food availability; however, the home physical activity environment did differ. White mothers reported greater access to indoor, outdoor, and neighborhood access to physical activity space and supports than Hispanic mothers, although effect sizes were small.

## 4. Discussion

This cross-sectional study compared the health and weight-related cognitions, behaviors, and home environments of mothers by race/ethnicity. A second purpose of this study was to identify specific topics to highlight in nutrition and obesity prevention interventions targeted to mothers of young children (Table 5). The proportion of White to non-White mothers in this study was similar to national averages; however, the proportion of Hispanics was higher (26% vs. 18%) and the proportion of Blacks was lower (8% vs. 13%) than the national averages [70]. This was likely due to targeted recruitment of Spanish-speakers and a higher proportion of Hispanics living in catchment areas (i.e., NJ and AZ, with 20% and 31% of the population being Hispanic, respectively) compared to the national average [71,72].

Participating mothers and their children had good health. Although maternal health did not differ by group, White mothers rated their children’s health higher than both Hispanic and Asian mothers. The reason for these differing ratings is not known. However, some research suggests that maternal acculturation level and/or maternal country of nativity may contribute to lower perceptions of child health [73,74]. Study findings indicate that future research should examine actual child health and factors affecting maternal perceptions of children’s health status.

With regard to the weight-related behaviors of study participants, all mothers tended to be physically inactive, which is congruent with national data [75,76,77,78]. Despite being less confident in their ability to promote healthy physical activity behaviors in children, modeling of physical activity less frequently, and having less space and supports for physical activity, Hispanic mothers reported their children had physical activity levels comparable to children in other groups. Space and supports for physical activity are strong positive predictors of physical activity [56,79,80,81,82,83], and thus could explain why Hispanic mothers had lower physical activity levels. However, the similarity of Hispanic children’s physical activity level to counterparts is incongruous with this explanation. Research indicates that space and support availability may not be perceived accurately. For instance, research in the southeastern United States found that the actual number of recreation facilities and miles of sidewalks available were similar in low- and high-income neighborhoods, despite respondent perceptions of variations based on socioeconomic status [84]. Study findings indicate that increasing awareness of already available supports for physical activity may be important to highlight as a means for increasing use of supports and physical activity levels.

Screentime for mothers was high, with Hispanic mothers engaging in more daily screentime than comparators. However, all mothers reported lower than amounts of screentime than comparable national data [85]. Children’s screentime also was high in all racial/ethnic groups and was comparable to national data [86,87], far exceeding the American Academy of Pediatrics recommendation of limiting screentime to 1 h or less per day [85,86,87,88,89,90] The excessive screentime of both mothers and children are likely due to home media environments replete with media devices [91,92]. Study findings indicate that it is critical to continue to encourage parents to limit their own as well as children’s use of media devices during leisure time and encourage and facilitate physical activity [88,90,93].

Mother’s dietary intake was congruent with dietary recommendations for fruits and vegetables in that they ate approximately 5 servings of these foods daily [94]. In contrast, few adults in the U.S. have adequate intake of fruits and vegetables (13% and 9%, respectively) [95,96]. Although it is not known why mothers’ intakes were higher than national data, it may be that mothers overreported intake [97], were influenced by self-report bias knowing that they were participating in a program to improve family health [98], or self-selected for participation in this study and may actually have been attracted to it because they wanted validation for their healthy behaviors [99,100,101,102,103]. However, mothers’ home environments had enough fruits and vegetables available for family members to eat the recommended 5 servings/day. Future nutrition and health promotions should continue to emphasize the value of adequate consumption of fruits and vegetables and strategies for meeting intake guidelines.

Sugar-sweetened beverage intake was low in study participants when compared to national averages [104]. National averages indicate Hispanic and Black adult women have a higher intake of calories from sugar-sweetened beverages than Whites and Asians, whereas no racial/ethnic differences were found in this study [104]. National averages show that beverage consumption patterns are changing in the U.S. so that children are consuming fewer sugar-sweetened beverages, less milk, and more water [105,106,107]. Children, especially those of Asian mothers in this study, had very low intakes of sugar-sweetened beverages. Like other studies, children of Hispanic and Black mothers consumed more sugar-sweetened beverages and fruit juice than other groups [108]; however, sugar-sweetened beverage consumption was still low and juice intake was within recommendations (less than 4–6 ounces of juice per day) [109]. Child milk intake was less than 1 cup daily for all racial/ethnic groups except Asian children. However, no group reached the recommended intake of approximately 2 cups/day for this age group [94]. Interventions aimed at mothers of young children should reinforce downward trends in sugar-sweetened beverage intake while simultaneously promoting milk or other rich sources of calcium to support optimal bone health of growing children.

The home food environment also serves a critical role in the development and support of dietary behaviors for both parents and children [110,111]. Many studies have reported differences in the home food environment by race/ethnicity. For instance, NHANES data showed that White families have a higher availability of salty snacks, soft drinks, and reduced fat milk [112]. Other research found that White households had healthier home food environments than other racial/ethnic groups [113] whereas another study showed that Hispanic families had greater availability of fruits, vegetables, and soft drinks than Black households [114,115]. Contrary to some studies, no differences were identified in the home food environment by maternal race/ethnicity [112,115]. The quantity of fruits/vegetables and sugar-sweetened beverages study participants had in their home likely helped participants to eat the recommended servings of fruits/vegetables and limit sugar-sweetened beverage intake [96,116]. By building on these healthful practices, future interventions can help family create a home food environment supportive of family health and obesity prevention.

Experts recommend that parents utilize non-controlling feeding practices (e.g., modeling healthy eating) and limit controlling feeding practices as they have been associated with excess weight gain and negative eating behaviors [117,118]. Mothers in this study tended to model healthy eating behaviors, with Asian mothers reporting the most modeling. Mothers also reported low to moderate use of negative feeding practices, and similar to previous research, Asian mothers tended to engage in these practices more. Asian, as well as Black, mothers exerted more control over children’s food intake amounts than comparators. Similar to previous research [119,120], Hispanic mothers in this study were more likely to pressure their children to eat. These racial/ethnic differences in child feeding practices could suggest a desire to encourage children to eat more, possibility due to food insecurity, cultural differences in parenting styles and/or body weight perceptions vis-à-vis health status, or other factors related to acculturation or socioeconomic status [119,120,121,122]. Study findings suggest the need to provide opportunities for parents to develop skills and motivation for implementing positive child feeding practices, with these opportunities being especially important in interventions targeting Asian and Hispanic mothers.

Although previous studies reported that Black households have family meals less often and Hispanic families share more family meals [114,123,124,125], all mothers in this study reported that they have frequent family meals with no racial/ethnic differences in family meal frequency. Few studies have examined racial/ethnic differences in family meal location; one study [126] reported Hispanic families and Asian families ate fewer meals outside of the home than White families [126]. Others found that Hispanic families ate more meals outside of the home than other racial/ethnic groups and Black families had a greater frequency of fast food meals than White families [127]. Within the home, Skala et al. reported that Hispanic families had fewer meals in front of the television compared to other racial/ethnic groups [114]. In this study, however, Hispanic mothers had family meals more frequently in locations associated with less nutrient dense meals (i.e., in the car, at a fast food restaurant, in front of the TV), and less frequently in healthy locations (i.e., the dining or kitchen table), perhaps suggesting less time available for preparing meals. With regard to importance placed on family meals, this study supports findings of others that no differences by race/ethnicity were noted [113]. Despite having differences in family meal location, mothers agreed that family meals are important and were confident in their ability to promote healthy eating in their children. Study findings indicate that future interventions should capitalize on the positive family meal cognitions and frequency behaviors of families and help parents, especially those who are Hispanic, develop strategies for increasing the rate at which family meals are eaten in situations associated with healthier meals.

Hispanic mothers differed from one or more groups on 18 of the 28 weight-related variables, with the most common differences being with White mothers. White mothers differed from at least one other racial/ethnic group on 17 weight-related variables, with differences most commonly with Hispanic mothers. Asian mothers differed from other mothers on 7 weight-related variables, with the most common differences being with Hispanic and White mothers. Black mothers also differed from others on just 7 of the weight-related variables and the differences were about equally divided among the other racial/ethnic groups. Although the cause of the differences, in particular the frequent differences in weight-related variables of Hispanic mothers—accounting for 24 of the total of 36 significant differences noted in weight-related variables—was beyond the scope of this study. However, it is important to note that although demographic characteristics differed, these differences had small effect sizes. In essence, with the exception of racial/ethnic group, the mothers in this study were demographically similar. All groups had education beyond high school, had moderate family affluence, and low food insecurity risk, and were in their early thirties. These similarities suggest factors beyond demographics are contributing to the differences observed. Home environments have been cited by others as a factor contributing to racial/ethnic differences [56,113,114]. In this study, the home food environment did not differ among groups, whereas the home physical activity environment did differ between White and Hispanic mothers. However, the effect size was very small. Findings seem to indicate the home environment contributed little to the differences observed. Future research should investigate other factors that may be contributing to the differences observed in Hispanic mothers vis-à-vis weight-related variables, such as cultural beliefs and acculturation level [128].

The findings of this study are limited by its cross-sectional, self-report nature, and potential for social desirability bias. Causal relationships cannot be determined due to the cross-sectional nature of this investigation. Strengths include the use of valid, reliable scales and a large, diverse sample of mothers of young children. In addition, unlike other studies exploring racial/ethnic differences, mothers in this study had similar demographic characteristics and home food and physical activity environments.

## 5. Conclusions

The results highlight key differences in health status and weight-related cognitions, behaviors, and home environments of mothers and their young children by race/ethnicity, especially between Hispanic and White mothers. The findings also suggest topics to highlight in nutrition and obesity prevention interventions that could improve the effectiveness of nutrition education interventions geared towards helping parents of young children develop healthier weight-related cognitions, behaviors, and home environments. Policies that support the widespread implementation of interventions, along with future research and monitoring of interventions implementing these topics, would provide evidence needed to determine their usefulness in ameliorating obesity. This study lends support to others suggesting that maternal race/ethnicity is important to consider in the design of nutrition education programs [129]. Additionally, study findings indicate further work is needed to explore the role that factors such as acculturation may have in influencing nutrition and physical activity cognitions and behaviors and how these factors can be used to informed the development of more effective nutrition interventions that are sensitive to racial/ethnic differences and appeal to the sensibilities of mothers.

## Figures and Tables

**Table 1:** Demographic Characteristics by Maternal Race/Ethnicity (*N* = 568).

Weight-Related Characteristics	White (*n* = 340) Mean ± SD (95% CI *)	Hispanic (*n* = 149) Mean ± SD (95% CI *)	Black (*n* = 46) Mean ± SD (95% CI *)	Asian (*n* = 33) Mean ± SD (95% CI *)	*F* *df = 3, 564 ^#^*	ANOVA *p* ^†^	Partial Eta-Squared
**Age**	33.33 ± 5.44 (32.75, 33.91)	30.73 ± 5.56 (29.83, 31.63)	33.37 ± 5.98 (31.59, 35.15)	34.73 ± 3.54 (33.47, 35.98)	9.801	<0.0001ADE	0.050
**Maternal Education ^1^**	2.39 ± 0.71 (2.31, 2.46)	2.15 ± 0.70 (2.04, 2.27)	2.20 ± 0.72 (1.98, 2.41)	2.73 ± 0.63 (2.51, 2.95)	7.972	<0.0001ACEF	0.041
**Family Affluence Score ^2^**	5.59 ± 1.73 (5.41, 5.78)	5.05 ± 1.82 (4.76, 5.35)	4.83 ± 1.62 (4.34, 5.31)	5.67 ± 1.36 (5.18, 6.15)	5.398	0.001AB	0.028
**Food Insecurity Risk ^3^**	1.72 ± 0.95 (1.62, 1.82)	1.89 ± 0.92 (1.74, 2.04)	1.91 ± 0.94 (1.63, 2.19)	1.61 ± 0.86 (1.30, 1.91)	1.819	0.142	0.010
**Parents in Household ^4^**	1.86 ± 0.35 (1.82, 1.90)	1.74 ± 0.44 (1.67, 1.81)	1.61 ± 0.49 (1.46, 1.76)	2.00 ± 0.00 (2.00, 2.00)	11.025	0.000ABEF	0.055
**Maternal Employment Status ^5^**	2.11 ± 0.90 (2.01, 2.20)	2.03 ± 0.94 (1.87, 2.18)	2.13 ± 0.88 (1.87, 2.39)	1.97 ± 0.92 (1.64, 2.30)	0.468	0.704	0.002

* CI = confidence interval; ^#^ df = degrees of freedom; ^†^ capital letters indicate Tukey post-hoc test significant (*p* < 0.05) differences between pairs: A: Whites vs. Hispanics; B: Whites vs. Blacks; C: Whites vs. Asians; D: Hispanics vs. Blacks; E: Hispanics vs. Asians; F: Blacks vs. Asians. ^1^ Education: high school or less, some college or associate degree, bachelor’s degree or higher; scored 1 to 3, respectively. ^2^ Family Affluence Scale contains 4 items: scores range from 0 to 9, and higher scores indicate greater family affluence. ^3^ Food Insecurity Risk scale: possible score range = 1 to 4, and higher scores indicate greater risk of food insecurity [1]. ^4^ Parents in Household: possible score range = 1 to 2. ^5^ Employment: possible score range = 1 to 3; 1 = does not work, 2 = works part time, and 3 = works full time.

**Table 2:** Maternal Weight-Related Characteristics by Race/Ethnicity (*N* = 568).

Weight-Related Characteristics	White (*n* = 340) Mean ± SD (95% CI *)	Hispanic (*n* = 149) Mean ± SD (95% CI *)	Black (*n* = 46) Mean ± SD (95% CI *)	Asian (*n* = 33) Mean ± SD (95% CI *)	*F* *df = 3, 564 ^#^*	ANOVA *p* ^†^	Partial Eta-Squared
**Maternal Health**							
Health Status ^1^	3.46 ± 0.96 (3.36, 3.56)	3.30 ± 0.91 (3.15, 3.45)	3.59 ± 1.02 (3.28, 3.89)	3.64 ± 0.82 (3.34, 3.93)	1.953	0.120	0.010
**Maternal Cognitions**							
Self-Efficacy for Promoting Child Physical Activity ^2^	3.57 ± 1.01 (3.46, 3.68)	3.40 ± 0.97 (3.25, 3.56)	3.91 ± 0.98 (3.61, 4.20)	3.69 ± 0.91 (3.37, 4.01)	3.305	0.020D	0.017
Self-Efficacy for Promoting Child Healthy Eating ^2^	3.80 ± 0.70 (3.73, 3.88)	3.56 ± 0.73 (3.44, 3.68)	3.94 ± 0.82 (3.70, 4.19)	3.78 ± 0.72 (3.53, 4.04)	5.168	0.002AD	0.027
**Maternal Behaviors**							
Physical Activity Level ^3^	14.74 ± 9.84 (13.69, 15.79)	12.38 ± 8.29 (11.03, 13.72)	13.22 ± 11.06 (9.93, 16.50)	15.55 ± 11.70 (11.40, 19.69)	2.433	0.064	0.013
Maternal Screentime (minutes/day)	329.47 ± 260.98 (301.63, 357.31)	411.34 ± 325.47 (358.65, 464.03)	377.28 ± 267.71 (297.78, 456.78)	261.36 ± 168.63 (201.57, 321.16)	4.369	0.005AE	0.023
Maternal Modeling of Physical Activity through Co-Play with Child (days/week) ^4^	3.83 ± 1.83 (3.63, 4.02)	3.15 ± 1.80 (2.86, 3.45)	3.42 ± 1.98 (2.84, 4.01)	3.42 ± 1.70 (2.82, 4.03)	4.947	0.002A	0.026
Fruit and Vegetable Intake (servings/day) ^5^	4.54 ± 1.84 (4.35, 4.74)	4.30 ± 1.78 (4.01, 4.59)	4.28 ± 2.19 (3.63, 4.93)	4.70 ± 2.14 (3.94, 5.46)	0.916	0.433	0.005
Sugar-Sweetened Beverage Intake (servings/day) ^5^	0.69 ± 0.86 (0.60, 0.78)	0.84 ± 0.78 (0.71, 0.97)	0.72 ± 0.76 (0.50, 0.95)	0.59 ± 0.71 (0.34, 0.84)	1.489	0.217	0.008
Maternal Modeling of Healthy Eating ^6^	3.69 ± 0.77 (3.61, 3.77)	3.46 ± 0.77 (3.34, 3.59)	3.59 ± 0.91 (3.32, 3.86)	3.83 ± 0.71 (3.58, 4.09)	3.726	0.011A	0.019
**Child Feeding Practices**							
Use Food to Reward Child’s Healthy Eating ^6^	2.34 ± 0.73 (2.26, 2.42)	2.39 ± 0.78 (2.26, 2.52)	2.35 ± 0.77 (2.12, 2.58)	2.79 ± 0.82 (2.50, 3.08)	3.576	0.014CE	0.019
Pressures Child to Eat ^6^	2.20 ± 0.95 (2.10, 2.30)	2.51 ± 0.90 (2.36, 2.66)	2.14 ± 0.97 (1.86, 2.43)	2.72 ± 1.06 (2.34, 3.09)	6.320	<0.0001ACF	0.033
Controls Child Food Amounts ^6^	3.05 ± 0.80 (2.96, 3.13)	3.19 ± 0.72 (3.07, 3.38)	3.60 ± 0.85 (3.35, 4.00)	3.70 ± 0.65 (2.98, 3.68)	12.828	<0.0001BCDE	0.064

* CI = confidence interval; ^#^ df = degrees of freedom. ^†^ Capital letters indicate Tukey post-hoc test significant (*p* < 0.05) differences between pairs: A: Whites vs. Hispanics; B: Whites vs. Blacks; C: Whites vs. Asians; D: Hispanics vs. Blacks; E: Hispanics vs. Asians; F: Blacks vs. Asians. ^1^ A 5-point agreement rating: poor, fair, good, very good, and excellent; scored 1 to 5, respectively; higher score indicates better health [2,3]. ^2^ A 5-point self-efficacy rating: not at all confident, not confident, confident, quite confident, and very confident; scored 1 to 5, respectively; scale score equals average of item scores; higher scale score indicates greater expression of the trait. Possible score range = 1 to 5. Cronbach alpha for the 3-item Self-Efficacy for Promoting Child Physical Activity scale = 0.81 and for the 6-item Self-Efficacy for Promoting Children’s Healthy Eating Behaviors scale = 0.77. ^3^ Days/week engaged in walking, moderate activity, and vigorous activity weighted by exercise intensity (weights of 1, 2, 3, respectively) and summed to create a scale score; higher scale score indicates greater activity level. Possible score range = 0 to 42 [4,5,6]. ^4^ Days/week mother engages in physical activity with child. Possible score range = 0 to 7; Cronbach alpha for this 3-item scale = 0.65. ^5^ Higher score indicates greater servings consumed daily [6,7,8,9,10,11]. ^6^ A 5-point agreement rating: strongly disagree, disagree, neither agree nor disagree, agree, strongly agree; scored 1 to 5, respectively; scale score equals average of item scores; higher scale scores indicate greater expression of the trait. Possible score range = 1 to 5. Cronbach alphas for the Models Healthy Eating, Uses Food to Reward Child’s Healthy Eating, Pressures Child to Eat, and Controls Child Food Intake Amounts scales are 0.71, 0.75, 0.66, and 0.66, respectively.

**Table 3:** Child Weight-Related Characteristics by Maternal Race/Ethnicity (*N* = 568).

Weight-Related Characteristics	White (*n* = 340) Mean ± SD (95% CI *)	Hispanic (*n* = 149) Mean ± SD (95% CI *)	Black (*n* = 46) Mean ± SD (95% CI *)	Asian (*n* = 33) Mean ± SD (95% CI *)	*F* *df = 3, 564 ^#^*	ANOVA *p* ^†^	Partial Eta-Squared
**Child Health Status ^1^**	4.51 ± 0.72 (4.43, 4.58)	4.15 ± 0.87 (4.01, 4.29)	4.37 ± 0.71 (4.16, 4.58)	3.94 ± 0.97 (3.60, 4.28)	10.980	<0.0001AC	0.055
**Child Physical Activity Behaviors**							
Child Physical Activity Level ^2^	26.50 ± 11.58 (25.26, 27.73)	25.05 ± 11.03 (23.27, 26.84)	25.85 ± 12.80 (22.05, 29.65)	24.00 ± 10.96 (20.11, 27.89)	0.873	0.455	0.005
Child Screentime (minutes/day)	283.24 ± 269.85 (254.45, 312.02)	337.55 ± 276.89 (292.73, 382.38)	282.39 ± 258.48 (205.63, 359.15)	254.09 ± 196.65 (184.36, 323.82)	1.769	0.152	0.009
**Child Beverage Intake (servings/day) ^3^**							
Sugar-Sweetened Beverages	0.28 ± 0.45 (0.24, 0.33)	0.39 ± 0.45 (0.32, 0.46)	0.45 ± 0.51 (0.30, 0.61)	0.17 ± 0.25 (0.08, 0.26)	4.625	0.003EF	0.024
Milk	0.84 ± 0.36 (0.80, 0.88)	0.77 ± 0.38 (0.70, 0.83)	0.70 ± 0.39 (0.58, 0.81)	1.04 ± 0.19 (0.97, 1.11)	7.424	<0.0001BCEF	0.038
100% Fruit Juice	0.53 ± 0.39 (0.49, 0.57)	0.67 ± 0.37 (0.61, 0.73)	0.70 ± 0.34 (0.60, 0.80)	0.50 ± 0.37 (0.37, 0.64)	6.440	<0.0001AB	0.033

* CI = confidence interval; ^#^ df = degrees of freedom. ^†^ Capital letters indicate Tukey post-hoc test significant (*p* < 0.05) differences between pairs: A: Whites vs. Hispanics; B: Whites vs. Blacks; C: Whites vs. Asians; D: Hispanics vs. Blacks; E: Hispanics vs. Asians; F: Blacks vs. Asians. ^1^ A 5-point agreement rating: poor, fair, good, very good, excellent; scored 1 to 5, respectively; higher score indicates better health [2,3]. ^2^ Days/week engaged in walking, moderate activity, and vigorous activity weighted by exercise intensity (weights of 1, 2, 3, respectively) and summed to create a scale score; higher scale score indicates greater activity level. Possible score range = 0 to 42 [4,5,6]. ^3^ Higher score indicates greater servings eaten daily [6,7,8,9,10,11].

**Table 4:** Home Environment Characteristics by Maternal Race/Ethnicity (*N* = 568).

Characteristic	White (*n* = 340) Mean ± SD (95% CI *)	Hispanic (*n* = 149) Mean ± SD (95% CI)	Black (*n* = 46) Mean ± SD (95% CI)	Asian (*n* = 33) Mean ± SD (95% CI)	*F* *df = 3, 564 ^#^*	*p* ^†^	Partial Eta-Squared
**Family Mealtime**							
Importance Placed on Family Meals ^1^	4.48 ± 0.63 (4.41, 4.54)	4.43 ± 0.68) (4.32, 4.54	4.32 ± 0.76 (4.09, 4.54)	4.20 ± 0.76 (3.93, 4.47)	2.319	0.074	0.012
Family Meal (meals/week)	12.88 ± 4.59 (12.39, 13.37)	11.85 ± 5.01 (11.04, 12.66)	11.15 ± 5.68 (9.47, 12.84)	13.03 ± 5.44 (11.10, 14.96)	2.882	0.035	0.015
**Family Meal Location (days** **/week)**							
Car	0.36 ± 1.02 (0.25, 0.47)	1.15 ± 2.15 (0.81, 1.50)	0.52 ± 1.24 (0.15, 0.89)	0.39 ± 1.00 (0.04, 0.75)	11.015	<0.0001ADE	0.055
Fast Food Restaurant	0.70 ± 1.08 (0.59, 0.82)	1.31 ± 1.45 (1.07, 1.54)	1.07 ± 1.58 (0.60, 1.54)	1.12 ± 1.58 (0.56, 1.68)	8.518	<0.0001A	0.043
Front of TV	1.90 ± 2.38 (1.65, 2.16)	2.84 ± 2.51 (2.43, 3.24)	3.02 ± 2.59 (2.25, 3.79)	2.39 ± 2.18 (1.62, 3.17)	6.857	<0.0001AB	0.035
Dining Table	5.08 ± 2.29 (4.83, 5.32)	4.05 ± 2.65 (3.62, 4.48)	4.43 ± 2.75 (3.62, 5.25)	4.88 ± 2.29 (4.07, 5.69)	6.448	<0.0001A	0.033
**Household Food Availability (servings/person/day) ^2^**							
Fruit/Vegetables	6.03 ± 2.03 (5.81, 6.25)	5.74 ± 2.21 (5.38, 6.09)	5.79 ± 2.12 (5.16, 6.41)	6.47 ± 1.42 (6.96, 6.97)	1.502	0.213	0.008
Sugar-Sweetened Beverages	0.22 ± 0.26 (0.19, 0.25)	0.26 ± 0.23 (0.22, 0.30)	0.27 ± 0.29 (0.19, 0.36)	0.27 ± 0.26 (0.18. 0.36)	1.507	0.212	0.008
**Home Physical Activity Environment**							
Indoor/Home Space and Supports for Physical Activity ^1^	3.39 ± 0.80 (3.31, 3.48)	3.11 ± 0.93 (2.96, 3.26)	3.46 ± 0.97 (3.18, 3.75)	3.27 ± 0.89 (2.95, 3.58)	4.306	0.005A	0.022
Outdoor/Yard Space and Supports for Physical Activity ^1^	4.45 ± 0.59 (4.39, 4.52)	4.20 ± 0.81 (4.06, 4.35)	4.39 ± 0.76 (4.14, 4.65)	4.15 ± 0.68 (3.89, 4.41)	5.108	0.002A	0.029
Neighborhood Space and Supports for Physical Activity ^1^	4.12 ± 0.99 (4.02, 4.23)	3.77 ± 1.02 (3.60, 3.93)	4.07 ± 1.05 (3.75, 4.38)	4.17 ± 0.65 (3.93, 4.40)	4.792	0.003A	0.025

* CI = confidence interval. ^#^ df = degrees of freedom; all are the same except for sugar-sweetened beverages df = 3, 563; Outdoor/Yard Space and Supports for Physical Activity df = 3, 507; and Neighborhood Space and Supports for Physical Activity df = 3, 556. ^†^ Capital letters indicate Tukey post-hoc test significant (*p* < 0.05) differences between pairs: A: Whites vs. Hispanics; B: Whites vs. Blacks; C: Whites vs. Asians; D: Hispanics vs. Blacks; E: Hispanics vs. Asians; F: Blacks vs. Asians. ^1^ A 5-point agreement rating: strongly disagree, disagree, neither agree nor disagree, agree, strongly agree; scored 1 to 5, respectively; scale score equals average of item scores; higher scale scores indicate greater expression of the characteristic. Possible score range = 1 to 5. Cronbach alpha for Importance Placed on Family meals = 0.63; Indoor/Home Space and Supports for Physical Activity = 0.71; Outdoor/Yard Space and Supports for Physical Activity = 0.74; Neighborhood Space and Supports for Physical Activity = 0.42. ^2^ Higher score indicates greater servings available daily per household member [6,7,8,9,10,11].

**Table 5:** Topics to Highlight in Nutrition and Obesity Prevention Interventions Targeted to Mothers of Young Children.

Key Topics for Nutrition and Obesity Prevention Interventions
Increase awareness of available physical activity supports to promote use of supports and physical activity.
Encourage parental limits on their own as well as children’s use of sedentary media devices during leisure time to facilitate physical activity.
Emphasize the value of adequate consumption of fruits and vegetables and strategies for meeting intake guidelines.
Reinforce downward trends in sugar-sweetened beverage intake.
Promote intake of milk or other rich sources of calcium.
Provide guidance for creating a home food environment supportive of family health and obesity prevention.
Provide opportunities for parents to develop skills and motivation for implementing positive child feeding practices; these opportunities may be especially important in interventions for Asian and Hispanic mothers.
Reinforce positive family meal cognitions and frequent family meals.
Promote strategies for increasing the frequency of eating family meals in locations associated with healthier meals; these strategies may be especially important for Hispanic mothers.
